# Clinical effectiveness of orange peel polymethoxy-flavonoids rich fraction as a palatal dressing material compared to Alveogyl: randomized clinical trial

**DOI:** 10.1038/s41598-024-53511-4

**Published:** 2024-02-06

**Authors:** Alzahraa A. Alghriany, Ahmed U. Ali, Iman S. A. Khallaf, Abeer S. Hassan, Marwa A. Sayed, Ahmed Mortada Fikry

**Affiliations:** 1 Department of Oral Medicine, Periodontology, and Diagnosis, Faculty of Dentistry, Assiut University, Assiut, Egypt; 2 Department of Pharmaceutics, Faculty of Pharmacy, Merit University, Sohag, Egypt; 3 Pharmacognosy and Natural Products Department, Faculty of Pharmacy, Menoufia University, Shibin Elkom, Egypt; 4 Department of Pharmaceutics, Faculty of Pharmacy, South Valley University, Qena, Egypt; 5 Department of Industrial Pharmacy, Faculty of Pharmacy, Assiut University, Assiut, Egypt

**Keywords:** Diseases, Health care, Medical research, Signs and symptoms

## Abstract

This study assessed the clinical effectiveness of orange peel polymethoxy-flavonoids rich fraction (OPMF) solid dispersion as a palatal dressing material, compared with Alveogyl, in a randomized clinical trial. After harvesting free gingival grafts for 18 patients in three groups, the donor site in group I received OPMF; group II received Alveogyl; and group III received placebo dough material. The visual analog scale (VAS) pain score in group I showed the lowest value in week one without a significant difference. In week 2, there was a substantial decrease in pain in group I compared to group III. Week 4 showed reduced pain scores in all groups without significant differences. The results of the number of analgesic pills revealed, after 1 week, the lowest number of pills consumed in group I, with a considerable difference compared to group III. Healing process results showed that group I had the highest healing values in each interval, with a significant difference between group I and group III at 1 and 2 weeks. Color matching parameter showed slight differences between the groups’ readings in favor of group I in all intervals without a statistically significant difference. The results suggest OPMF as a palatal dressing material that facilitates hemostasis, pain relief, and palatal wound healing.

## Introduction

In periodontal surgery, the hard palate is the best place for harvesting a free gingival graft (FGG). Palatal grafts, due to their superior clinical outcomes and autogenous nature, are preferred above other allogenic or synthetic grafts^1^.

Achieving widened keratinized and attached gingiva, deepening the vestibular depth, covering exposed root surfaces, and changing a thin periodontal phenotype to a thick phenotype are some clinical endpoints associated with the long-standing free gingival graft procedure^2,3^. These endpoints contribute to efficient primary stability, which is essential for healing^4^.

After the FGG has been harvested from the donor site, the open wound takes 2 to 4 weeks to heal with primary or secondary intention^5^. 3 to 5 weeks are typically needed for complete epithelialization^6,7^.

This healing process progresses through four distinct but overlapping stages: hemostasis, inflammation, granulation, and maturation^8^. It’s worth noting that wounds in the mouth heal and re-epithelialize more quickly than skin wounds^9,10^.

The patient morbidity at the palatal donor site is the principal drawback of the FGG treatment^11^. The most common side effects of FGG harvesting include discomfort, pain, and bleeding at the donor site, potentially affecting a patient’s quality of life, including speech, eating, and drinking problems^12^.

Assessing the patients’ perception of their treatment is essential to reducing patient discomfort. Various methods have been reported to decrease postoperative pain^13^.

Therefore, a variety of substances have been investigated for their effects on the palatal donor site, including stents, periodontal packs, growth factors^14^, absorbable gelatin sponges, absorbable collagen dressings^15^, hyaluronic acid (HA), Alveogyl, low-level laser therapy^16^, and medical plant extracts^11^.

Flavonoids are secondary metabolic plants with a wide variety of potential biological activities. They are polyphenolic compounds that do not contain nitrogen in their chemical structure. Polymethoxy-flavones, a subcategory of flavonoids isolated from citrus peel, are renowned for their potential biological functions, such as analgesics^17^, anti-inflammatory, antibacterial, and antioxidant activities, which are required for successful wound healing^18,19^.

This study utilized orange peel extract rich in polymethoxy-flavonoids; despite their beneficial characteristics, their aqueous solubility and oral bioavailability are limited. Additionally, the extract’s consistency resembles a highly viscous exudate, which hinders its pharmaceutical application. Hence, a delivery system that enhances solubility and permeability and offers a localized application for healing palatal wounds after FGG harvesting is deemed necessary^20^.

Solid dispersion is one of the most attractive techniques for enhancing the dissolution of poorly soluble drugs, where the lipophilic drug is dispersed in a hydrophilic carrier in different ways. The final product is characterized by minimized particle size, enhanced wettability, and solubility^20^.

Alveogyl, a topical combination of natural substances, is frequently used to effectively treat alveolar osteitis and decrease pain and infection^21,22^. Alveogyl is a brown fibrous dressing applied topically to prevent dry sockets following extraction. Its active components include iodoform, an iodine-based antibacterial agent^23^; butamben, an ester local anesthetic; and eugenol, an essential oil with obvious pain-reduction capabilities. Vegetable fibers from the Penghawar djambi plant, which have hemostatic qualities, carry these active components^24^. Consequently, it can be used as a dressing material^25^.

However, a few studies have suggested that Alveogyl might extend wound healing in alveolar osteitis treatment. Additionally, three reported cases indicated that Alveogyl caused an unexplained foreign body reaction^26,27^. Therefore, evaluating novel natural materials and comparing their effectiveness to the widely used Alveogyl is necessary. The present study aimed to clinically compare, for the first time, the effects of orange peel polymethoxy-flavonoids rich fraction (OPMF) versus Alveogyl as a palatal wound dressing or no dressing material on the severity of postoperative pain, amount of analgesic consumed, palatal wound healing, and tissue color matching following free gingival graft harvesting in a randomized controlled clinical trial.

## Methods

### Ethical approval

The Research Ethics Committee of the Faculty of Medicine, Assiut University, approved this prospective randomized control trial. This trial was conducted following the ethical principles outlined in the Declaration of Helsinki, with IRB permission number 17300948, and it has been registered on clinicaltrial.gov with the ID: NCT05814003 since April 14, 2023. The study was performed and reported according to CONSORT 2010 guidelines.

### Eligibility criteria

This study was conducted at the Faculty of Dentistry, Assiut University, Egypt. Informed consent was obtained from each patient after the procedure was explained.

Adult, healthy patients aged 18 years or older were enrolled with keratinized gingiva ≤ 1 mm (evaluated with a UNC periodontal probe) and needed free gingival grafts for various periodontal and peri-implant plastic surgeries. Patients were excluded based on the following criteria: (1) Smoking; (2) Pregnancy or breastfeeding; (3) Systemic diseases that interfere with wound healing, such as diabetes mellitus, immunodeficiency, radiation, metabolic disorders, or immunosuppressive drugs; (4) Use of anti-inflammatory drugs or narcotic analgesics within the past three months; (5) Individuals who have undergone palatal grafting procedures at the same site in the past.

### Study design

A three-arm, parallel randomized clinical trial was conducted using simple randomization. Sealed envelopes with numbered cards from 1 to 18 were employed for allocation, with the distribution as follows: cards 1 to 6 for Group I, cards 7 to 12 for Group II, and cards 13 to 18 for Group III, maintaining an allocation ratio of 1:1:1. Two different wound dressing materials were applied to the palatal donor following free gingival graft harvesting, dividing the participants into three groups: Group I received orange peel polymethoxy-flavonoids rich fraction (OPMF) dressing material, Group II received Alveogyl dressing material, and Group III received placebo material.

### Sample size and characteristics

**Figure 1:**
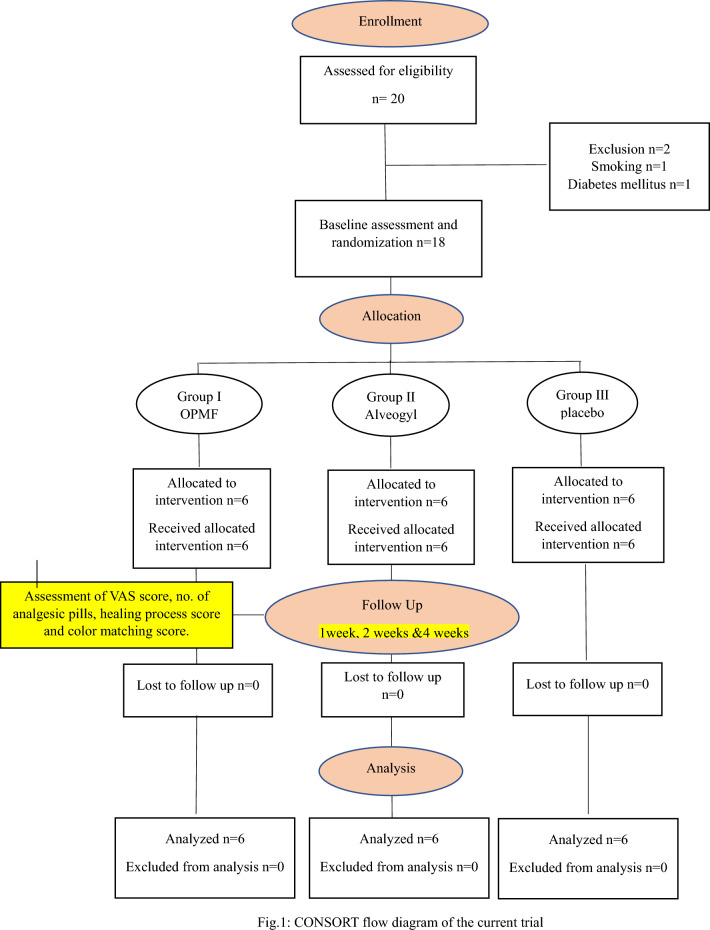
CONSORT flow diagram of the current trial.

Based on a prior study^11^ and calculation using the G power statistical power analysis tool (version 3.1.9.4)^28^ for sample size calculation, detection of large effect size (f) = 0.88 requires a total sample size of n = 18, divided into n = 6 in each group. The analysis assumes an actual power (1-error) of 0.8 (80%) and a significance level (error) of 0.05 (5%) for a two-sided hypothesis test (see Fig. 1).

### Orange peel polymethoxy-flavonoids rich fraction dough preparation

The orange peel extract was prepared following the method described by Khallaf et al.^29^. Orange peel was air-dried at room temperature, powdered (10 g), and subjected to extraction by maceration using dichloromethane (50 ml × 3). The extract was concentrated under a vacuum to remove solvent and volatile oil. The resulting solid residue was kept at − 10 °C until the time of the experiments. The solid dispersion of the prepared extract was then prepared using β-cyclodextrin through the co-grinding technique, maintaining a 3:1 ratio of β-cyclodextrin to orange peel extract^30^.

The placebo material is formed only from β-cyclodextrin, a non-active ingredient used as a carrier or inert additive. Its purpose is to increase the bioavailability of active substances and decrease the concentration of active compounds in the final product without compromising their effectiveness^31^.

### Surgical procedure

**Figure 2:**
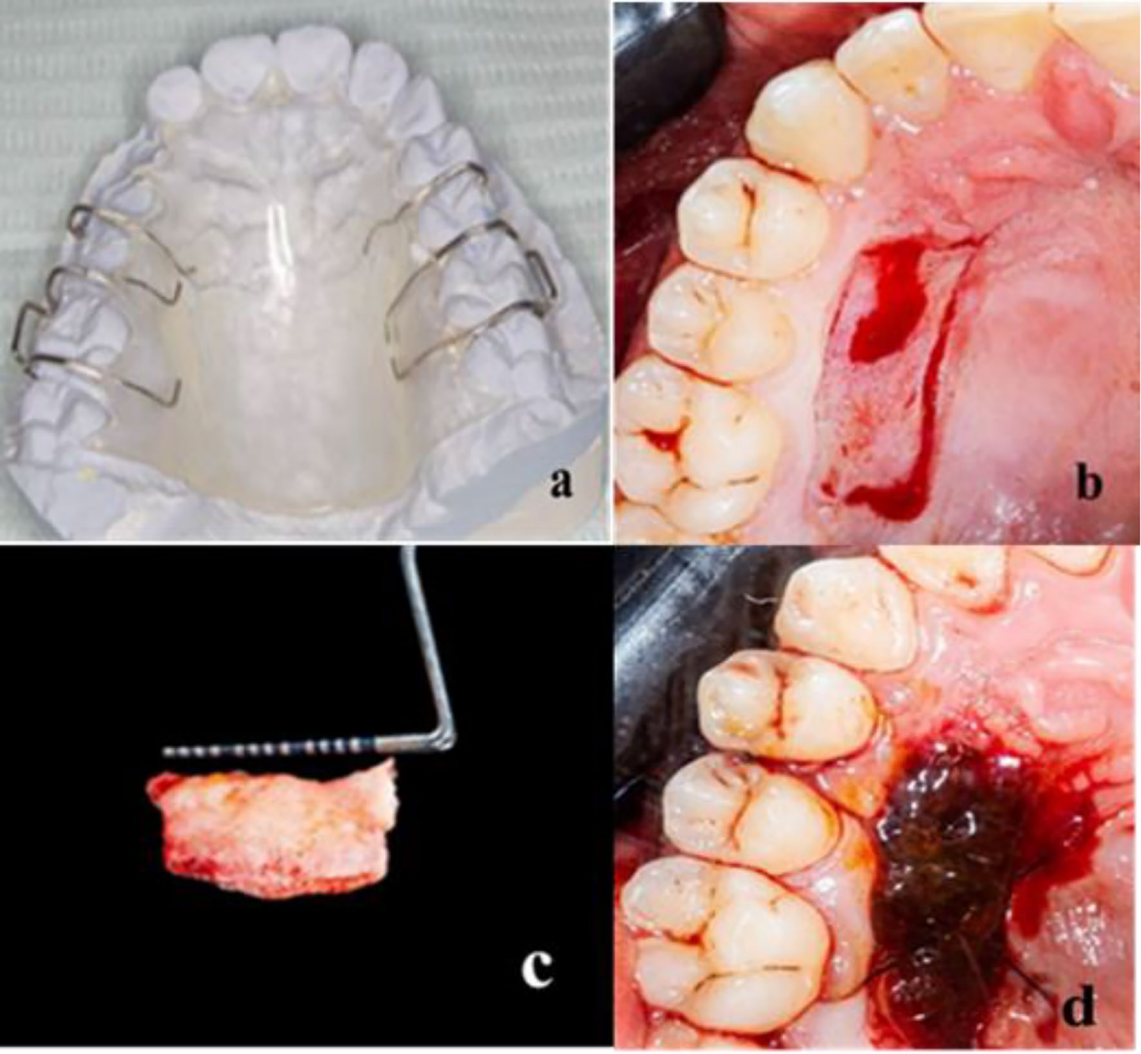
(**a**) A surgical stent; (**b**) FGG dimensions after the use of the template; (**c**) FGG before suturing to the recipient area; (**d**) Orange peel polymethoxy-flavonoids rich fraction (OPMF) dressing material placement to the donor site and suturing.

A surgical stent was prepared to protect the donor area by taking an impression of the palatal region before surgery, as shown in Fig. 2a. The stent’s fit was examined before the surgery.

The recipient and palatal donor surgical sites received local anesthesia (4% articaine and 0.001% adrenalin). Additionally, the recipient site underwent the first stage of surgical preparation for the FGG.

A sterile template was utilized to estimate the FGG’s dimensions. A conventional scalpel technique was employed to harvest a 1.0–1.5 mm split-thickness gingival graft (Fig. 2b) from the palatal mucosa adjacent to the premolars and the first molar, positioned 2–3 mm apical to the gingival margin of neighboring teeth.

The graft (Fig. 2c) was placed in the recipient area, firmly adjusted, and stabilized with knotted sutures. Additionally, the recipient site received a gentle compress for 5 min using gauze soaked in saline.

The donor site in the first group received orange peel polymethoxy-flavonoids rich fraction (OPMF) dressing material (Fig. 2d); the donor site in the second group received Alveogyl (Septodont, Niederkassel, Germany) dressing material; and the donor site in the third group received placebo dough material. Subsequently, all groups underwent suturing with resorbable material and the insertion of an acrylic stent.

The participants were not informed about the kind of applied material they would receive, and the tested materials were all the same color.

Following the operation, postoperative instructions were provided to each patient. They were advised to adhere to a soft diet and take one Ibuprofen 600 mg tablet every eight hours on the first postoperative day and then as needed based on the severity of the pain. Additionally, patients were instructed to use a mouthwash containing 0.12 percent chlorhexidine twice daily.

The primary outcome of this trial was the assessment of pain, while the secondary outcomes included the evaluation of the healing process, analgesics consumed, and color matching.

### Patient assessment

#### Subjective assessment

- *Pain assessment* The visual analog scale (VAS) measured the patient’s pain level^32^. During this procedure, patients were asked to rate their pain level on a scale from 0 to 10, where 0 = no pain, 1–3 = mild, 4–6 = moderate, and 7–10 = severe pain.
- *Total analgesics taken* The number of Ibuprofen 600 mg pills needed to control postoperative pain during the 14 days following surgery was recorded.

#### Objective assessment

- *The healing process* The Landry, Turnbull, and Howley Healing Index (HI) were used for the evaluation^33,34^. This index assigns a value from 1 (very poor healing) to 5 (great healing) based on criteria such as redness, hemorrhage, granulation tissue, epithelialization, and suppuration.
- *Color matching* The Modified Manchester Scar Scale^35^ was used to categorize color matching concerning adjacent mucosa into three categories: 1—a perfect match, 2—a minor mismatch, and 3—an evident mismatch.

### Follow-up

After surgery, patients were offered follow-up appointments in the first, second, and fourth weeks to collect assessment data.

### Statistical analysis

Clinical data were statistically analyzed at 1 week, 2 weeks, and one month using a paired t-test and SPSS (Statistical Package for Social Sciences) software. The Kruskal–Wallis test was employed for comparing all studied groups each week, the Mann–Whitney test for comparisons between two groups in each week, Friedman’s test for comparing all weeks within each group, and the Wilcoxon Signed Ranks test for comparing different weeks within each group. Statistical significance was considered at a p-value of < 0.05.

## Results

This study involved 18 participants randomly assigned to three groups, with six participants in each group. Among the participants, 11 (61.1%) were females, and 7 (38.9%) were males.

Regarding the VAS of pain, at 1 week postoperative, the highest pain score was presented in the placebo group (Group III) of 5.5 ± 1.64, indicating moderate pain. Group II, which received Alveogyl, showed a pain score of 4.33 ± 1.21, indicating moderate pain. The orange peel polymethoxy-flavonoids rich fraction (OPMF) group (Group I) had the lowest value of 3.83 ± 0.75, representing moderate pain. However, the different groups had no significant difference in pain scores (p = 0.157).

At 2 weeks postoperative, the pain score was mild in Group I (1.67 ± 0.52), mild in Group II (2 ± 0.89), and moderate in Group III (3.17 ± 0.75). There was a statistically significant difference between Group I and Group III (p < 0.01) and between Group II and Group III (p < 0.05).

At 4 weeks postoperative, the pain score was very mild in Group I and Group II (0.33 ± 0.52), mild in Group III (0.83 ± 0.98), and there was no statistically significant difference in pain scores among different groups (p = 0.558).

**Figure 3:**
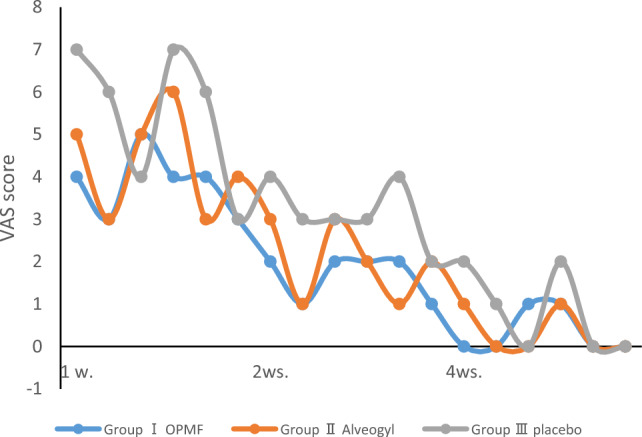
Statistical comparison of 4 weeks of VAS readings among the three groups.

Intragroup comparisons showed statistically significant differences within each group across different intervals (p < 0.05), as illustrated in Table 1 and Fig. 3.

**Table 1:** VAS presented as range (min.–max.), mean ± SD (standard deviation), and Median (IQ range) (interquartile range) evaluated along the follow-up visits for orange peel polymethoxy-flavonoids rich fraction (OPMF) group (Group I) (n = 6), Alveogyl group (Group II) (n = 6) and placebo group (Group III) (n = 6).

VAS							
	Group I (n = 6)	Group II (n = 6)	Group III (n = 6)	p-value1	p1	p2	p3
1 week							
Range	3–5	3–6	3–7				
Mean ± SD	3.83 ± 0.75	4.33 ± 1.21	5.5 ± 1.64				
Median IQ range	4 (3–4.25)	4.5 (3–5.25)	6 (3.75–7)	0.157	0.452	0.084	0.165
2 weeks							
Range	1–2	1–3	2–4				
Mean ± SD	1.67 ± 0.52	2 ± 0.89	3.17 ± 0.75				
Median IQ range	2 (1–2)	2 (1–3)	3 (2.75–4)	0.018*	0.484	0.007**	0.044*
4 weeks							
Range	0–1	0–1	0–2				
Mean ± SD	0.33 ± 0.52	0.33 ± 0.52	0.83 ± 0.98				
Median IQ range	0 (0–1)	0 (0–1)	0.5 (0–2)	0.558	1.000	0.367	0.367
p-value2	0.002**	0.002**	0.002**				
p4	0.020*	0.020*	0.027*				
p5	0.024*	0.026*	0.026*				
p6	0.023*	0.026*	0.026*				

p-value1: For comparing all studied groups each week, use the Kruskal–Wallis test.

p1: for comparing orange peel polymethoxy-flavonoids rich fraction & Alveogyl groups each week by Mann–Whitney test.

p2: comparing orange peel polymethoxy-flavonoids rich fraction & placebo groups each week by Mann–Whitney test.

p3: for comparing Alveogyl and placebo groups each week by Mann–Whitney test.

p-value2: for comparing all weeks within each group by Friedman’s test.

p4: for comparing between 1 and 2 weeks at each group by Wilcoxon Signed Ranks test.

p5: comparing 1 week and 4 weeks at each group by Wilcoxon Signed Ranks test.

p6: for comparing 2 weeks and 4 weeks each group by Wilcoxon Signed Ranks test.

*Statistically significant at p < 0.05.

**Statistically significant at p < 0.01.

The number of analgesic pills results after 1 week postoperative revealed that Group III had the highest values (18.17 ± 2.79), showing a significant difference compared to Group I (13.33 ± 3.01) (p < 0.05). Group II had a value of 16.83 ± 3.13, with no statistically significant difference from both other groups.

**Figure 4:**
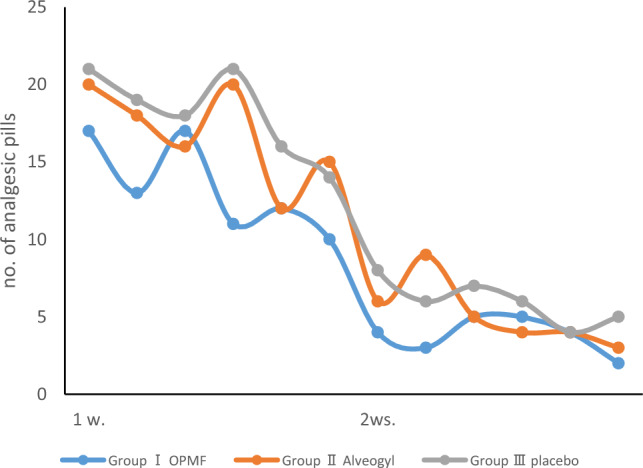
Statistical comparison of the number of analgesic pills consumed within 2 weeks among the three groups.

At 2 weeks, the highest value was observed in Group III (6 ± 1.41), followed by Group II (5.17 ± 2.14), while the lowest value was found in Group I (3.83 ± 1.17). Intragroup comparisons showed statistically significant differences within each group across different intervals (p < 0.05), as demonstrated in Table 2 and Fig. 4.

**Table 2:** Number of pills presented as range (min.–max.), mean ± SD (standard deviation), and Median (IQ range) (interquartile range) evaluated along the follow-up visits for orange peel polymethoxy-flavonoids rich fraction (OPMF) group (Group I) (n = 6), Alveogyl group (Group II) (n = 6), and placebo group (Group III) (n = 6).

No. of pills							
	Group I (n = 6)	Group II (n = 6)	Group III (n = 6)	p-value1	p1	p2	p3
1 week							
Range	10–17	12–20	14–21				
Mean ± SD	13.33 ± 3.01	16.83 ± 3.13	18.17 ± 2.79				
Median IQ range	12.5 (10.75–17)	17 (14.25–20)	18.5 (15.5–21)	0.054	0.091	0.024*	0.420
2 weeks							
Range	2–5	3–9	4–8				
Mean ± SD	3.83 ± 1.17	5.17 ± 2.14	6 ± 1.41				
Median IQ range	4 (2.75–5)	4.5 (3.75–6.75)	6 (4.75–7.25)	0.078	0.285	0.023*	0.290
p-value2							
p4	0.027*	0.028*	0.027*				
p5							
p6							

p-value1: For comparing all studied groups each week, use the Kruskal–Wallis test.

p1: for comparing orange peel polymethoxy-flavonoids rich fraction & Alveogyl groups each week by Mann–Whitney test.

p2: comparing orange peel polymethoxy-flavonoids rich fraction & placebo groups each week by Mann–Whitney test.

p3: for comparing Alveogyl and placebo groups each week by Mann–Whitney test.

p4: for comparing 1 week and 2 weeks at each group by Wilcoxon Signed Ranks test.

*Statistically significant at p < 0.05.

**Statistically significant at p < 0.01.

**Figure 5:**
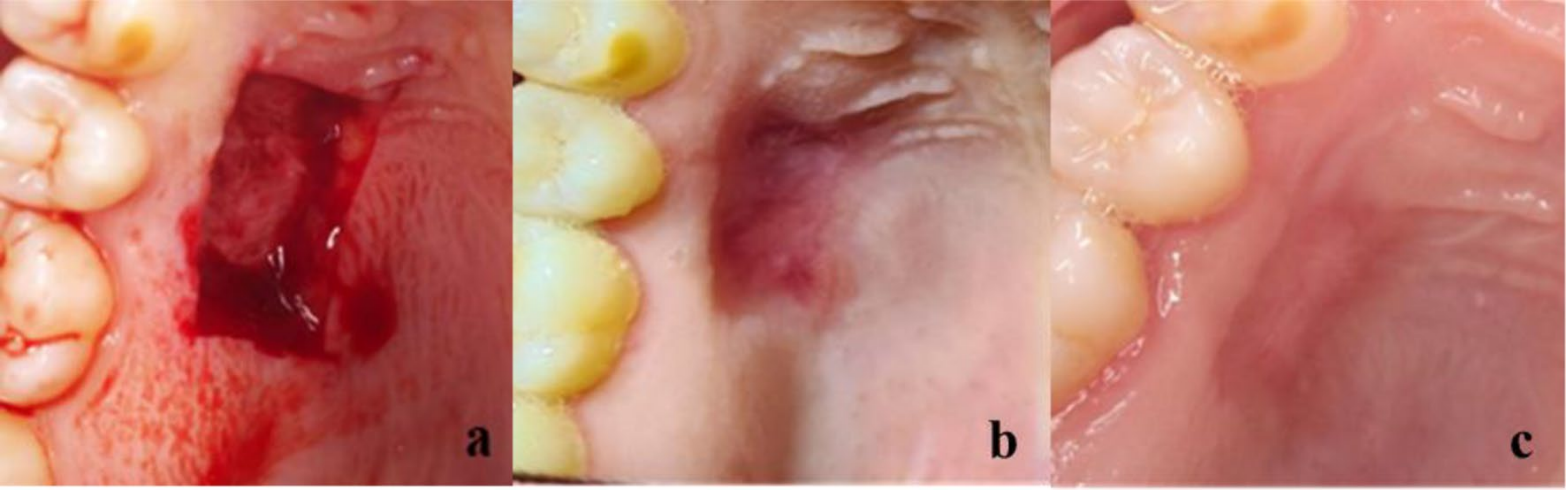
Clinical photographs show the FGG donor site in Group I postoperatively before application of OPMF (**a**), after 1 week (**b**), and after 4 weeks (**c**).

**Figure 6:**
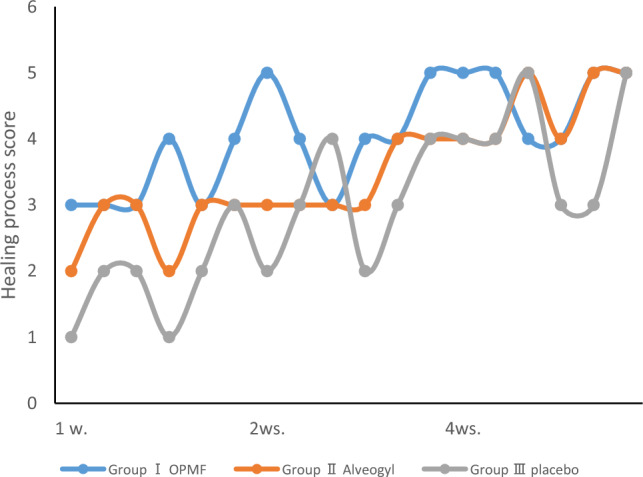
Statistical comparison of 4 weeks’ healing process scores among the three groups.

The healing process results showed that Group I had the highest healing values in each interval. At 1 week and 2 weeks, there was a significant difference between Group I (3.33 ± 0.52, 4.17 ± 0.75) and Group III (1.83 ± 0.75, 3 ± 0.89) (p < 0.01 and p < 0.05, respectively). At 4 weeks, the highest value was found in Group I (4.67 ± 0.52), followed by Group II (4.50 ± 0.55), while the lowest value was found in Group III (4 ± 0.89). The three groups had no statistically significant difference, as presented in Table 3 and Figs. 5 and 6.

**Table 3:** Healing process presented as range (min.–max.), mean ± SD (standard deviation), and Median (IQ range) (interquartile range) evaluated along the follow-up visits for orange peel polymethoxy-flavonoids rich fraction (OPMF) group (Group I) (n = 6), Alveogyl group (Group II) (n = 6), and placebo group (Group III) (n = 6).

Healing process							
	Group I (n = 6)	Group II (n = 6)	Group III (n = 6)	p-value1	p1	p2	p3
1 week							
Range	3–4	2–3	1–3				
Mean ± SD	3.33 ± 0.52	2.67 ± 0.52	1.83 ± 0.75				
Median IQ range	3 (3–4)	3 (2–3)	2 (1–2.25)	0.008**	0.056	0.007**	0.057
2 weeks							
Range	3–5	3–4	2–4				
Mean ± SD	4.17 ± 0.75	3.33 ± 0.52	3 ± 0.89				
Median IQ range	4 (3.75–5)	3 (3–4)	3 (2–4)	0.063	0.057	0.044*	0.484
4 weeks							
Range	4–5	4–5	3–5				
Mean ± SD	4.67 ± 0.52	4.5 ± 0.55	4 ± 0.89				
Median IQ range	5 (4–5)	4.5 (4–5)	4 (3–5)	0.312	0.575	0.162	0.299
p-value2	0.014*	0.004**	0.003**				
p4	0.059	0.046*	0.020*				
p5	0.038*	0.020*	0.026*				
p6	0.083	0.020*	0.034*				

p-value1: For comparing all studied groups each week, use the Kruskal–Wallis test.

p1: for comparing hydroxylated poly methoxy flavones and Alveogyl groups each week by Mann–Whitney test.

p2: Comparing the hydroxylated polymethoxy flavones placebo group each week using the Mann–Whitney test.

p3: for comparing Alveogyl and placebo groups each week by Mann–Whitney test.

p-value2: for comparing all weeks within each group by Friedman’s test.

p4: Comparing 1 week and 2 weeks at each group by Wilcoxon Signed Ranks test.

p5: Comparing 1 week and 4 weeks at each group by Wilcoxon Signed Ranks test.

p6: Comparing two and 4 weeks for each group using the Wilcoxon Signed Ranks test.

*Statistically significant at p < 0.05.

**Statistically significant at p < 0.01.

**Figure 7:**
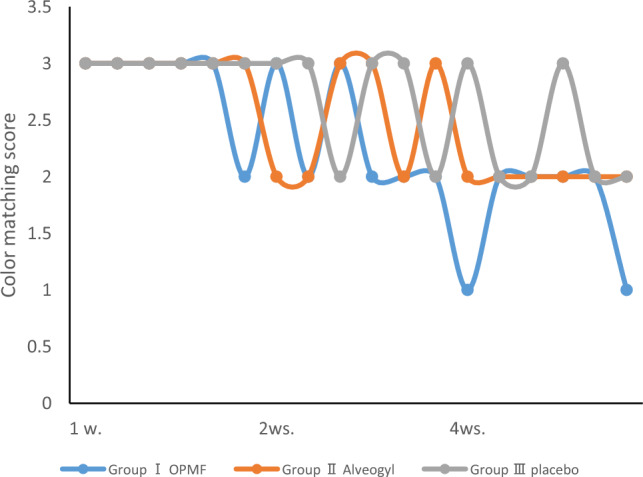
Statistical comparison of color-matching scores within 4 weeks among the three groups.

The color matching parameter showed a slight difference in reading between Group I (2.89 ± 0.41, 2.33 ± 0.52, 1.67 ± 0.52) in the first, second, and fourth weeks and both Group II (3, 2.5 ± 0.55, 2) and Group III (3, 2.67 ± 0.52, 2.33 ± 0.52). However, there was no statistically significant difference among the three groups, as shown in Table 4 and Figs. 5 and 7.

**Table 4:** Color matching presented as range (min.–max.), mean ± SD (standard deviation), and Median (IQ range) (interquartile range) evaluated along the follow-up visits for orange peel polymethoxy-flavonoids rich fraction (OPMF) group (Group I) (n = 6), Alveogyl group (Group II) (n = 6), and placebo group (Group III) (n = 6).

Color matching							
	Group I (n = 6)	Group II (n = 6)	Group III (n = 6)	p-value1	p1	p2	p3
1 week							
Range	2–3	3–3	3–3				
Mean ± SD	2.83 ± 0.41	3 ± 0	3 ± 0				
Median IQ range	3 (2.75–3)	3 (3–3)	3 (3–3)	0.368	0.317	0.317	1.000
2 weeks							
Range	2–3	2–3	2–3				
Mean ± SD	2.33 ± 0.52	2.5 ± 0.55	2.67 ± 0.52				
Median IQ range	2 (2–3)	2.5 (2–3)	3 (2–3)	0.533	0.575	0.269	0.575
4 weeks							
Range	1–2	2–2	2–3				
Mean ± SD	1.67 ± 0.52	2 ± 0	2.33 ± 0.52				
Median IQ range	2 (1–2)	2 (2–2)	2 (2–3)	0.059	0.138	0.056	0.138
p-value2	0.011*	0.011*	0.050				
p4	0.083	0.083	0.157				
p5	0.020*	0.014*	0.046*				
p6	0.102	0.083	0.157				

p-value1: For comparing all studied groups each week, use the Kruskal–Wallis test.

p1: for comparing orange peel polymethoxy-flavonoids rich fraction and Alveogyl groups each week by Mann–Whitney test.

p2: comparing orange peel polymethoxy-flavonoids rich fraction and placebo groups each week by Mann–Whitney test.

p3: for comparing Alveogyl and placebo groups each week by Mann–Whitney test.

p-value2: for comparing all weeks within each group by Friedman’s test.

p4: Comparing 1 week and 2 weeks at each group by Wilcoxon Signed Ranks test.

p5: Comparing 1 week and 4 weeks at each group by Wilcoxon Signed Ranks test.

p6: Comparing 2 and 4 weeks for each group using the Wilcoxon Signed Ranks test.

*Statistically significant at p < 0.05.

**Statistically significant at p < 0.01.

## Discussion

The most common issues after FGG procedures include postoperative bleeding, pain, and discomfort at the donor site^36^.

Various dressing materials have been recommended to preserve the resulting partial-thickness wound at the palatal donor site, enhance comfort, support the process of re-epithelialization, and protect the palatal connective tissue from physical and chemical irritation as well as colonization by oral microorganisms already present^37^.

Applying a topical dressing to the palatal wound allows for the local concentration of healing-promoting, analgesic, and antiseptic substances. At the same time, the risks of side effects or sensitization associated with systemic administration were reduced^24^.

Orange peel polymethoxy-flavonoids (OPMF) are natural and safe extracts^38^ from the flavonoid family, playing a significant role in wound healing in several ways. First, they exhibit an antioxidant action by inhibiting reactive oxygen species (ROS), thereby minimizing oxidative stress and accelerating wound healing^39^. In a previous study performed using a mouse model, it was found that treatment of diabetic foot ulcers with the flavonoid hesperidin resulted in complete healing of the wound within less than 21 days. This effect may be attributed to the enhancement of the expression of the Vascular Endothelial Growth Factor (VEGF-c), Angiopoietin-1 (Ang-1)/Tie-2, and Transforming Growth factor (TGF), leading to accelerated angiogenesis and stimulating new tissue restoration^40,41^. Flavonoids activated the expression of nuclear factor erythroid 2-related factor 2 (Nrf2), reducing oxidative stress and promoting cell proliferation, neovascularization, and wound healing. Additionally, Nrf2 activation inhibits cytoprotective genes, thereby upgrading keratinocyte apoptosis. Flavonoids also exert their analgesic and anti-inflammatory action by inhibiting the expression of nuclear factor kappa B (NF-kB), thereby minimizing the levels of inflammatory mediators such as prostaglandin E2 (PGE2), leukotriene B4 (LTB-4), interleukin 1 (IL-1), tumor necrosis factor (TNF-), interleukin 6 (IL-6), and interferon (IFN-)^42^. During injury, the commensal bacteria colonize the wound, forming a biofilm that postpones the healing process and makes the wound susceptible to new invasion. The antibacterial action of flavonoids was mediated in different ways, including blocking microbial adhesion and growth through complex action with the microorganism’s cell wall. Additionally, flavonoids mediate bacterial enzyme inhibition, such as tyrosyl‐tRNA synthetase. Baicalein, a flavonoid, when combined with cefotaxime, forms a powerful bactericide that minimizes the Pseudomonas aeruginosa‐induced secretion of the inflammatory cytokines (IL‐1β, IL‐6, IL‐8, and TNFα), which are essential for inflammatory injury after infection with *P. aeruginosa*^42^.

The active chemicals in Alveogyl include butamben, iodoform, and eugenol. Additionally, it contains olive oil, spearmint oil, sodium lauryl sulfate, calcium carbonate, penghawar djambi, and purified water^23^.

Iodoform is an iodine-based antibacterial, while butamben is an ester local anesthetic^24^. Eugenol, an essential oil extracted from various plants, including cloves, possesses exceptional pain-relieving qualities^43^. Alveogyl consistency is provided by the penghawar djambi, a byproduct of Cibotium barometz tree fibers^44,45^, which also offers hemostatic properties and ensures easy adherence to soft tissues in the correct dimensions^26^.

The current study used orange peel polymethoxy-flavonoids (OPMF) and Alveogyl as palatal wound dressing for a palatal wound following free gingival grafting.

Different objective measurements, including index and scales, wound epithelialization tests, visual clinical healing assessments, photographic healing, bleeding evaluations, cytological analyses^46,47^, laboratory analyses^48^, and histological examination^49^, were employed as methods to evaluate the outcomes of postoperative palatal wound healing^50^.

Pain perception is one of the most widely discussed techniques for evaluating FGG operations. The patients use the visual analog scale to express their perception^51^. Sousa et al.^15^ and others^52,53^ used the VAS-10 scale, ranging from 0 (no pain) to 10 (the worst suffering ever experienced).

In addition to evaluating the pain, Zucchelli et al.^54^ and other authors^55^ used the number of analgesics consumed in hours, days, or weeks to indicate the pain levels.

This study assessed postoperative pain directly via VAS and indirectly via analgesics. Patients received 600 mg of Ibuprofen on the day of surgery for pain control. Patients were instructed to take analgesic medications only when necessary to ensure that reported pain scores were attributed to the intervention adopted^54^.

The highest pain VAS score was observed in the placebo group (Group III) at 1 week postoperative, representing moderate pain. The Alveogyl group (Group II) showed moderate pain. The hydroxylated polymethoxy flavones group (Group I) had the lowest value, representing moderate pain, with no significant difference among the different groups.

By the second week, pain severity decreased in all the groups, becoming mild in both Group I and Group II and moderate in Group III, with a statistically significant difference between Group III and the other two groups. By the fourth week, as epithelialization increased and pain decreased, the differences between the groups decreased, and the values were no longer statistically different.

Although the number of analgesic tablets used for pain management by the patients in each group noticeably decreased over 14 days, those in Group III used a significantly higher number of analgesic tablets than the intervention Group I and Group II throughout the 14-day healing period.

Our results align with Ferraz et al.^56^, who approved the analgesic effect of flavonoids, and Ehab et al.^25^, who reported a significant reduction in VAS pain scores in the Alveogyl group.

All groups experienced more pain over the first week, gradually subsiding over the subsequent days. This trend is consistent with the findings of Del Pizzo^57^, who noted an increase in pain during the first 2 weeks following surgery.

According to Burkhardt et al.^5^, pain was more severe in the early postoperative days, subsiding over the next few days.

A Healing Index (HI) was proposed by Landry et al.^34^ to describe the extent of clinical healing after periodontal surgery, assessing the quality of the healing process. The HI, ranging from 1 (very poor) to 5 (excellent), combines the presence or absence of five clinical criteria (tissue color, response to palpation, granulation tissue, incision margin, and suppuration)^58^.

The healing process results showed that Group I had the highest healing values at each interval, with a significant difference between Group I and Group III at 1 week and 2 weeks, consistent with Zulkefli et al.^19^, who reported the wound-healing capacity of flavonoids.

Complete epithelialization of the palatal wound occurred 4 weeks after FGG surgery, according to Del Piezzo et al.^57^. Consistent with this research, most patients’ palatal lesions were fully healed in our study within 4 weeks.

Silva et al.^59^ reported that in most patients (92%), the palatal FGG donor site had completely epithelized and closed by 15 days after surgery.

Comparisons of the palatal donor site with adjacent and opposite sides were conducted by Bahammam et al.^14^ and others^53^ through visual inspection of clinical images, considering color match (CM) characteristics. Samani et al.^35^ used the Modified Manchester Scale to compare the color of the neighboring mucosa, in which 0 represented a perfect match, 1 indicated a slight mismatch, and 2 signified an obvious mismatch. The degree of reepithelization and wound healing will be reflected in the visible color changes when the FGG healing occurred by secondary intention and matched the surrounding normal tissue^35^.

Color Match (CM) was evaluated in the present study on days 7, 14, and 30. In the first, second, and fourth weeks, the color matching parameter showed a slight difference between Group I and Group II and Group III, with no statistically significant difference among the three groups.

The present trial shared common limitations, including subjective methods for quantifying donor healing, the absence of histology evaluation, and the wide variety and drawbacks of scoring systems.

## Conclusion

Within its limitations, the study suggests that using orange peel polymethoxy-flavonoids rich fraction (OPMF) as a wound dressing material, comparable to Alveogyl, may represent a suitable option to improve patients’ healing process and reduce postoperative pain.

## Data Availability

The data used in the current study are not publicly available due to ethical restrictions but are available from the corresponding author on reasonable request.
